# Novel mutation in the γ-S crystallin gene causing autosomal dominant cataract

**Published:** 2009-03-04

**Authors:** Vanita Vanita, Jai Rup Singh, Daljit Singh, Raymonda Varon, Karl Sperling

**Affiliations:** 1 Centre for Genetic Disorders, Guru Nanak Dev University, Amritsar, India; 2 Dr. Daljit Singh Eye Hospital, Amritsar, India; 3 Institute of Human Genetics, Charitè, University Medicine of Berlin, Berlin, Germany

## Abstract

**Purpose:**

To identify the underlying genetic defect in a north Indian family with seven members in three-generations affected with bilateral congenital cataract.

**Methods:**

Detailed family history and clinical data were recorded. Linkage analysis using fluorescently labeled microsatellite markers for the already known candidate gene loci was performed in combination with mutation screening by bidirectional sequencing.

**Results:**

Affected individuals had bilateral congenital cataract. Cataract was of opalescent type with the central nuclear region denser than the periphery. Linkage was excluded for the known cataract candidate gene loci at 1p34–36, 1q21–25 (gap junction protein, alpha 8 [*GJA8*]), 2q33–36 (crystallin, gamma A [*CRYGA*], crystallin, gamma B [*CRYGB*], crystallin, gamma C [*CRYGC*], crystallin, gamma D [*CRYGD*], crystallin, beta A2 [*CRYBA2*]), 3q21–22 (beaded filament structural protein 2, phakinin [*BFSP2*]), 12q12–14 (aquaporin 0 [*AQP0*]), 13q11–13 (gap junction protein, alpha 3 [*GJA3*]), 15q21–22, 16q22–23 (v-maf musculoaponeurotic fibrosarcoma oncogene homolog [*MAF*], heat shock transcription factor 4 [*HSF4*]), 17q11–12 (crystallin, beta A1 [*CRYBA1*]), 17q24, 21q22.3 (crystallin, alpha A [*CRYAA*]), and 22q11.2 (crystallin, beta B1 [*CRYBB1*], crystallin, beta B2 [*CRYBB2*], crystallin, beta B3 [*CRYBB3*], crystallin, beta A4 [*CRYBA4*]). Crystallin, alpha B (*CRYAB*) at chromosome 11q23–24 was excluded by sequence analysis. However, sequencing the candidate gene, crystallin, gamma S (*CRYGS*), at chromosome 3q26.3-qter showed a heterozygous c.176G→A change that resulted in the replacement of a structurally highly conserved valine by methionine at codon 42 (p.V42M). This sequence change was not observed in unaffected family members or in the 100 ethnically matched controls.

**Conclusions:**

We report a novel missense mutation, p.V42M, in *CRYGS* associated with bilateral congenital cataract in a family of Indian origin. This is the third report of a mutation in this exceptional member of the β-/γ-crystallin superfamily and further substantiates the genetic and clinical heterogeneity of autosomal dominant cataract.

## Introduction

Congenital cataract is a clinically and genetically heterogeneous group of eye disorders that causes visual impairment and childhood blindness. It accounts for 10%–30% of blindness in children [1]. Nearly one-third of the cases show a positive family history. Although all three Mendelian modes of inheritance have been observed for congenital cataract, autosomal dominant seems to be the most common mode of inheritance in non-consanguineous families [2,3].

At least 34 loci and mutations in 22 genes have been reported to be linked with different forms of congenital cataract. These genes encode for crystallins (crystallin, alpha A [*CRYAA*; OMIM 123580], crystallin, alpha B [*CRYAB*; OMIM 123590], crystallin, beta A1 [*CRYBA1*; OMIM 123610], crystallin, beta A4 [*CRYBA4*; OMIM 123631], crystallin, beta B1 [*CRYBB1*; OMIM 600929], crystallin, beta B2 [*CRYBB2*; OMIM 123620], crystallin, beta B3 [*CRYBB3*; OMIM 123630], crystallin, gamma C [*CRYGC*; OMIM 123680], crystallin, gamma D [*CRYGD*; OMIM 123690], crystallin, gamma S [*CRYGS*; OMIM 123730]), gap junctional molecules (gap junction protein, alpha 3 [*GJA3*; OMIM 121015] and *GJA8* [OMIM 600897]), lens major intrinsic proteins (major intrinsic protein of lens fiber [*MIP*; OMIM 154050] and lens intrinsic membrane protein 2 [*LIM2*; OMIM 154045]), beaded structural filament proteins (beaded filament structural protein 1, filensin [*BFSP1*; OMIM 603307] and *BFSP2* [ OMIM 603212]), regulatory factors (paired box gene 6 [*PAX6*; OMIM 607108], paired-like homeodomain 3 [*PITX3*; OMIM 602669], *HSF4* [OMIM 602438], *MAF* [OMIM 177075]), glucosaminyl (N-acetyl) transferase 2 (*GCNT2* [OMIM 600429]), chromatin modifying protein-4B (*CHMP4B* [OMIM 610897]), and transmembrane protein (*TMEM114* [OMIM 611579]).

We came across a three-generation north Indian family with individuals affected by bilateral congenital cataract at the Dr. Daljit Singh Eye Hospital (Amritsar, India). Using fluorescently labeled microsatellite markers, linkage was excluded to already known candidate gene regions at chromosome 1p, 1q, 2q, 3q21, 12q, 13q, 15q21, 16q, 17q, 17q24, 21q22.3, and 22q. *CRYAB* at chromosome 11q23–24 was excluded by sequencing. Upon sequence analysis of the candidate gene, *CRYGS*, localized at chromosome 3q26.3-qter, a novel sequence change (c.176G→A) was observed that resulted in the substitution of a highly conserved valine by methionine at codon 42 (p.V42M). This change co-segregated completely with the disease phenotype and was observed neither in unaffected family members nor in the 100 ethnically matched controls.

## Methods

### Family description

**Figure 1:**
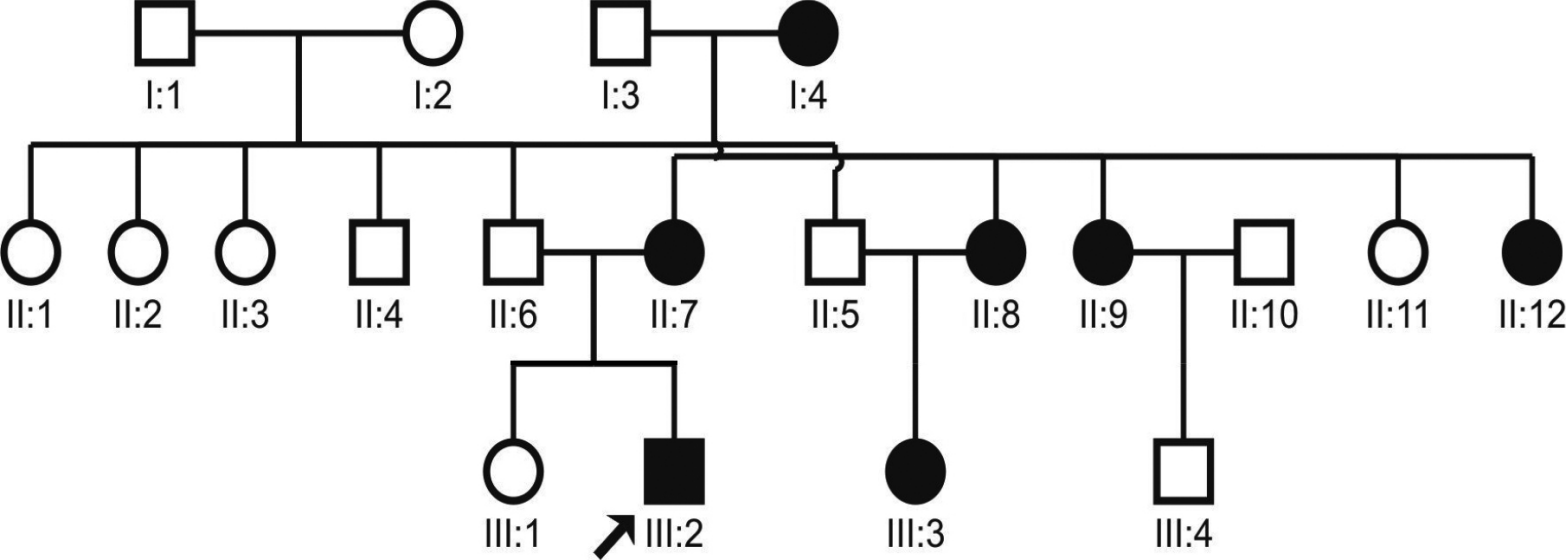
Pedigree of an Indian family with autosomal dominant congenital cataract (ADCC) is shown. The proband (III:2) is indicated by an arrow. Filled squares and circles represent affected males and females, respectively. Seven members in three generations were affected with bilateral cataract since childhood. Except the proband (III:2), all other affected members (I:4, II:7, II:8, II:9, II:12, III:2, III:3) were operated for bilateral congenital cataract in the first decade of their life.

**Figure 2:**
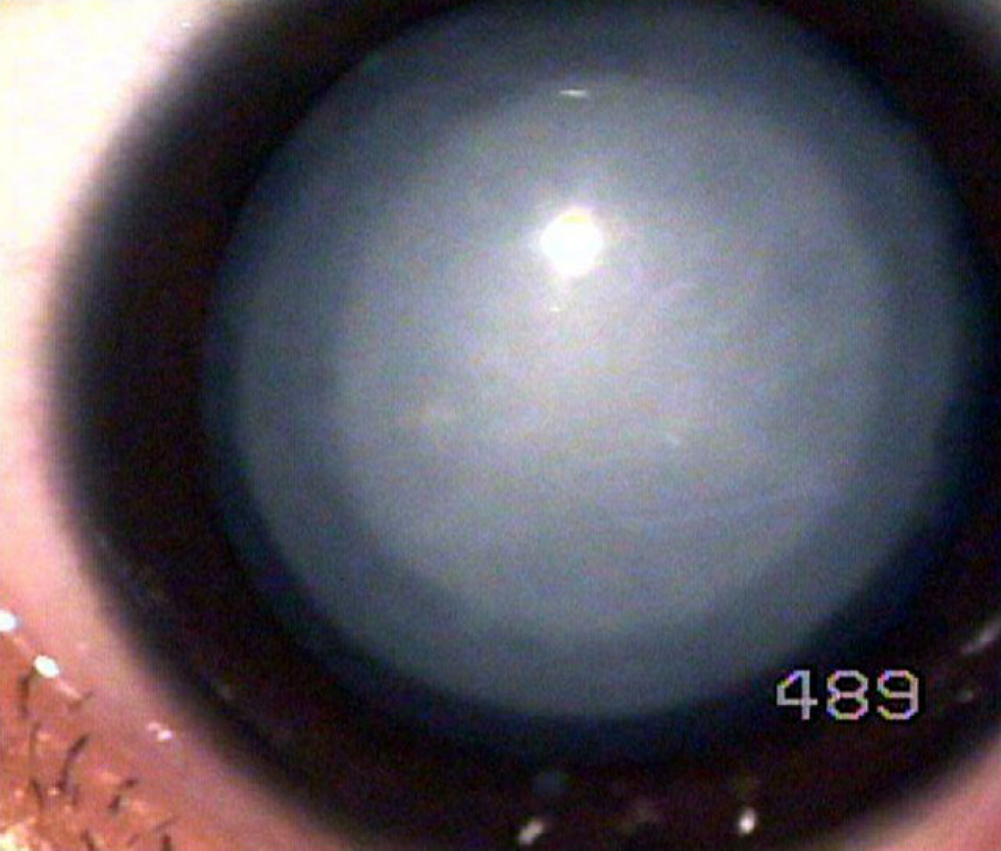
Slit lamp photograph of affected lens. A slit lamp photograph is shown of the affected lens (left eye) in the three-year-old proband (III:2, Figure 1). Cataract is of the opalescent type. The central nuclear region is more dense than the periphery.

The proband, a three-year-old child, was diagnosed with bilateral cataract. Family history revealed seven affected members in three generations (Figure 1). A detailed ophthalmic examination, including slit lamp examination on dilated pupil, was performed on 14 members of the family. It revealed that seven members including the proband (III:2) were bilaterally affected. Six individuals (I:4, II:7, II:8, II:9, II:12, and III:3) had a history of cataract extraction in childhood, and seven individuals (I:3, II:5, II:6, II:10, II:11, III:1, and III:4) were unaffected. The phenotype in the non-operated proband (III:2) was of opalescent type with the central nuclear region denser than the periphery (Figure 2). The cataract type was similar in both eyes of the proband except the left eye was more affected than the right eye. The opacities were visible in early childhood, and all the affected individuals underwent cataract surgery in the first decade of their life.

### Genotyping and linkage analysis

Informed consent was obtained from all individuals studied. This study was approved by the Ethics Review Board of the Guru Nanak Dev University (Amritsar, Punjab), consistent with the provisions of the Declaration of Helsinki. Blood was drawn and DNA was extracted by standard protocols. Linkage analysis based on semi-automated genotyping was performed with microsatellite markers on DNA samples of 14 clinically examined individuals (seven affected and seven unaffected including four spouses). Microsatellite markers and their distances were from the Marshfield linkage map. Seventy-five microsatellites from candidate gene regions at 1p34–36, 1q21–25 (*GJA8*), 2q33–36 (*CRYGA*, *CRYGB*, *CRYGC*, *CRYGD*, *CRYBA2*), 3q21–22 (*BFSP2*), 12q12–14 (*AQP0*), 13q11–13 (*GJA3*), 15q21–22, 16q22–23 (*MAF*, *HSF4*), 17q11–12 (*CRYBA1*), 17q24, 21q22.3 (*CRYAA*), and 22q11.2 (*CRYBB1*, *CRYBB2*, *CRYBB3*, *CRYBA4*) were analyzed. Microsatellites were amplified by touchdown polymerase chain reaction (PCR) using fluorescently labeled primers as described elsewhere [4]. PCR products were pooled, mixed with loading dye containing internal size standards, denatured at 95 °C for 5 min, and electrophoresed on 4% denaturing polyacrylamide gels on a DNA sequencer (ABI-Prism 377; ABI, Foster City, CA). Data were collected and analyzed by GENESCAN version 3.1.2 (ABI), and genotyping was done using GENOTYPER 2.5.1 software (ABI). Autosomal dominant inheritance with complete penetrance of the trait and a disease gene frequency of 0.0001 were assumed. Recombination frequencies (θ) were considered to be equal between males and females. Two-point linkage analysis was performed by MLINK from the LINKAGE program package [5], and multipoint analysis was undertaken using GENEHUNTER [6].

### Mutation analysis

The candidate genes, *CRYAB* (NM_001885) and *CRYGS* (NM_017541), which were localized at 11q23–24 and 3q26.3-qter, were analyzed by bidirectional sequence analysis. The primers were designed to amplify the coding regions and 30–50 bp flanking intronic regions using the PrimerSelect program of the Lasergene package (DNA STAR Inc., Madison, WI). Amplification and sequencing were performed as described elsewhere [4]. The data were assembled and analyzed using the SeqMan II program of the Lasergene package.

## Results

### Linkage analysis

The already known candidate gene regions for congenital cataract at 1p34–36, 1q21–25, 2q33–36, 3q21–22, 12q12–14, 13q11–13, 15q21–22, 16q22–23, 17q11–12, 17q24, 21q22.3, and 22q11.2 were tested using 75 fluorescently labeled microsatellite markers. Two-point LOD score values of less than −2.0 at θ=0.001 were obtained with all these markers, excluding these loci to be linked with cataract in this family.

### Mutation analysis

**Figure 3:**
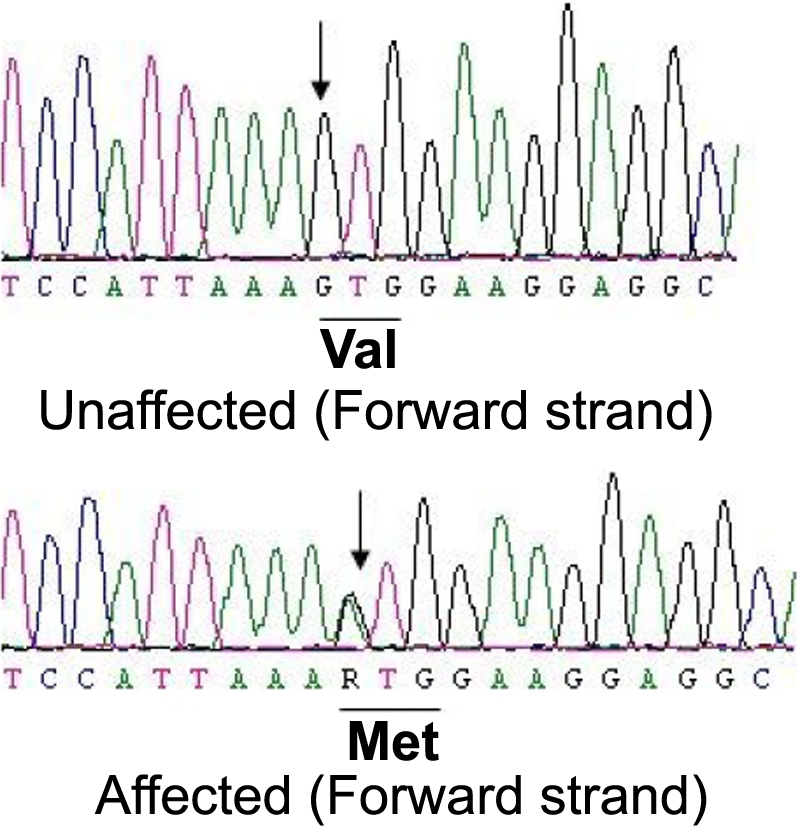
Mutation analysis of *CRYGS* in an unaffected and an affected individual. The wild-type G in the sequence of the unaffected individual (II:6; forward strand) and the heterozygous c. 176G→A change resulting in the substitution of valine-42 by methionine (p.V42M) in the affected individual (III:2; forward strand) are indicated by arrows.

The candidate genes, *CRYAB* (11q23–24) and *CRYGS* (3q26.3-qter), were analyzed by sequence analysis. Bidirectional sequence analysis of the coding regions of *CRYAB* in affected individuals (I:4, II:7, II:8, II:9, II:12, III:2, and III:3) did not show any sequence alteration, excluding *CRYAB* to be associated with cataract in this family. However, bidirectional sequence analysis of *CRYGS* indicated a novel heterozygous c.176G→A change (Figure 3) in all seven affected members (I:4, II:7, II:8, II:9, II:12, III:2, and III:3) of this family. This sequence alteration was not observed in any of the unaffected individuals (I:3, II:5, II:6, II:10, II:11, III:1, and III:4) or in the 100 ethnically matched normal controls (200 chromosomes). This nucleotide substitution replaces an evolutionarily highly conserved valine with methionine at amino acid position 42 (p.V42M).

## Discussion

Crystallins alpha (α-), beta (β-), and gamma (γ-) are the major structural proteins in the vertebrate eye lens, constituting approximately 90% of water soluble proteins. Their short range spatial order packing and stability are crucial to maintain lens transparency [7,8]. Crystallins are classified into two families, α-crystallins and the β-/γ-crystallin superfamily [9]. β- and γ-crystallins share a common two domain structure composed of four “Greek-key” motifs [10]. In humans, six γ-crystallin genes are organized as a cluster on 2q33–35. In contrast, *CRYGS* is located at 3q26.3-qter and characterized by an additional α helix, which is not found in the other crystallins [11]. γ-Crystallin shows the highest internal symmetry of any protein, and this property is assumed to be a major contributor to its stability [12]. They are localized mainly in the nuclear region of the lens. In lenses of individuals less than two years of age, the fraction of γ-crystallins has been reported to be 35% CRYGS, 45% CRYGC, and 20% CRYGD [13].

**Figure 4:**
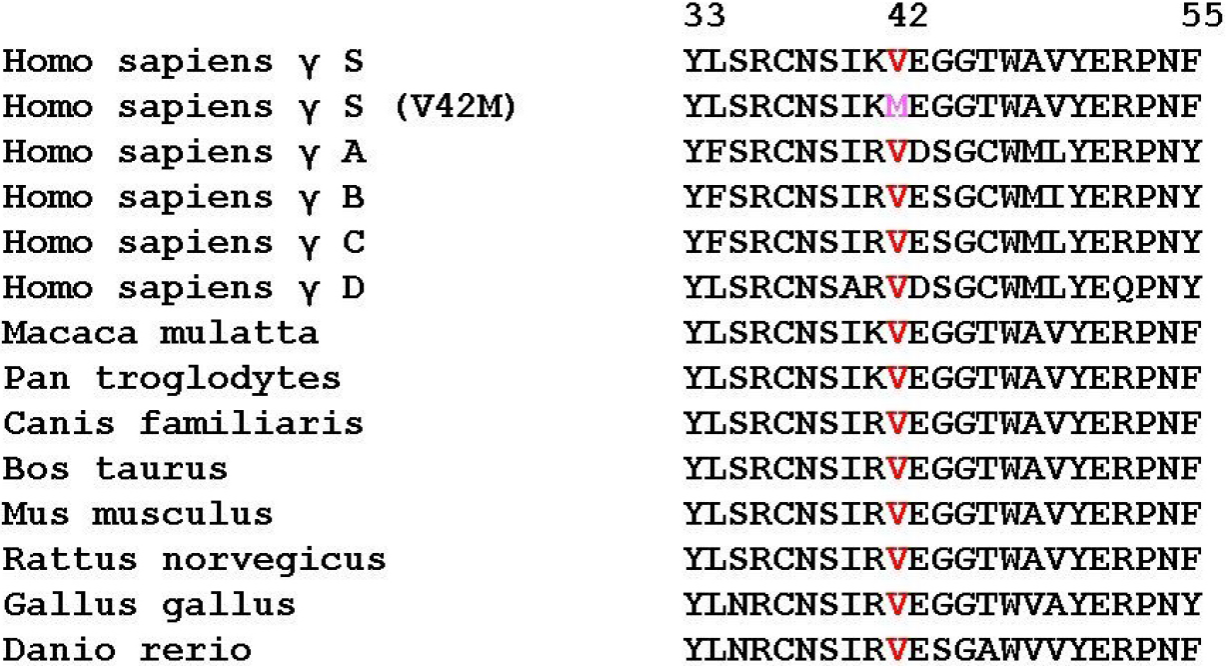
Amino acid sequence alignment of CRYGS. Multiple alignment of partial amino acid sequences of CRYGS from different *Homo sapien* γ crystallins (γ S, γ A, γ B, γ C, γ D) and from different species indicate that valine at position 42 (red) is highly conserved. The valine to methionine substitution at codon 42 (p.V42M) in γ S in the proband’s (III:2) sequence is also highlighted (pink).

In the present autosomal dominant congenital cataract (ADCC) family with seven affected members in three generations, 12 known candidate gene loci harboring 17 candidate genes were excluded by linkage analysis using fluorescently labeled microsatellite markers. In addition, due to the unavailability of microsatellite markers with us at 11q23–24 and 3q26.3-qter and the availability of primers for sequence analysis of candidate genes, *CRYAB* and *CRYGS*, at these respective loci, these genes were sequenced bidirectionally. No sequence alteration was found in the coding region of *CRYAB* in patients when compared to unaffected family members, thus excluding its association with ADCC in the present family. However, we observed a novel p.V42M substitution in the CRYGS polypeptide associated with bilateral congenital cataract. The p.V42M substitution is likely to cause cataract since it segregated with the phenotype and was not detected in either the unaffected family members or the 100 ethnically matched controls. The valine at codon 42 is highly conserved in different γ-crystallins (Figure 4).

The present study is the third report of a mutation in *CRYGS* linked with cataract (Table 1). Previously, Sun et al. [14] reported a p.G18V substitution in a Chinese family with 14 members in six generations affected by progressive polymorphic cortical cataract with cataractous changes prominent in affected older individuals and considerable variability in the phenotype. The anterior, posterior, and peripheral cortical regions showed opacities but not the fetal nuclear region [14]. Recently, Devi et al. [15] have reported a novel missense mutation (p.S39C) in a south Indian family with four members in two generations affected by sutural cataract with significant phenotypic variability concerning both size and density of the opacities. The phenotype of individual III:2 (Figure 3) differs from that of the Chinese family as the cataract is of opalescent type with the center nuclear region denser than the periphery in both affected lenses. It also differs from the south Indian family as no sutural opacities were observed. Cataract onset in affected individuals was in early childhood. Since all other affected individuals of this family were already operated in the first decade of their life, it was not possible to analyze the phenotypic variability.

**Table 1:** Identified mutations in human *CRYGS* associated with different types of congenital cataract.

**Amino acid change**	**Location**	**Cataract type/phenotype description**	**Origin of the family**	**Reference**
p.G18V	Exon 2 Motif 1; Domain 1	Autosomal dominant progressive cortical cataract showing opacities in the anterior, posterior and peripheral cortical regions. No opacity in the fetal nuclear region. Intra-familial and intra-ocular phenotypic variability observed in affected individuals.	Chinese	[14]
p.S39C	Exon 2 Motif 1; Domain 1	Autosomal dominant progressive juvenile onset cataract. Unilateral sutural opacities in one affected individual, lamellar opacities in another affected member. Phenotypic variation in the size, density and position of opacities documented for other affected individuals as well.	South Indian	[15]
p.V42M	Exon 2 Motif 1; Domain 1	Autosomal dominant congenital cataract. In the index case opalescent type cataract. Central nuclear region denser as compared to periphery. All other affected members were operated for bilateral cataract in their childhood.	North Indian	Present study

Reported mutations in human *CRYGS* associated with different types of congenital cataract are given. All three mutations (p.G18V in the Chinese family [14], p.S39C in the South Indian family [15], and p.V42M in the present north Indian family) are localized in domain 1 of CRYGS. Phenotypic variability has been observed in all these ADCC families.

Mutations in *CRYGS* have been reported to be associated with mice cataractogenesis as well. Sinha et al. [16] have reported a p.Phe9Ser substitution linked with inherited semi-dominant progressive cataract whereas Bu et al. [17] reported that p.Trp163Stop in *CRYGS* lead to recessive nuclear cataract.

Mutations in the γ-crystallins tend to produce nuclear or zonular cataract consistent with their high level of expression in the lens nucleus, although the phenotype may vary significantly as documented in various reports. Similarly, mutations in BFSP2 are being linked with different phenotypes like juvenile-onset progressive cataract [18] and pulverulent and spoke-like cortical opacities from birth [19]. At least four different mutations in CRYGC (T5P [Coppock-like cataract], GlyfsX62 [zonular pulverulent cataract], R168W [lamellar/nuclear cataract], and C109X [nuclear cataract]) have been reported to be associated with different phenotypes. Similarly, five different mutations in CRYGD (R14C [juvenile-onset pulverulent cataract], P23T [cerulean type and lamellar cataract], R36S [prismatic type cataract], R58H [aceuliform cataract], and W156X [nuclear cataract]) have been reported to be linked with entirely different phenotypes. Functional assay of R36S and R58H indicate that these mutations do not alter the protein fold but alter the surface characteristics of CRYGD, which lowers its solubility and enhances the crystal nucleation rate of these mutants and their precipitation in at least one case, forming crystals in the lens [20]. On the other hand, R14C in CRYGD makes it more susceptible to thiol-mediated aggregation [21].

More than a dozen mutations in GJA8 have been linked with different cataract phenotypes in different families with diverse mutational mechanisms like a dominant negative effect for p.P88S and p.P88Q [22-24] or a loss of function for p.D47A, p.G22R, and p.R23T [25-27]. Even identical mutations like Q155X in CRYBB2 lead to different cataract phenotypes. The phenotypic differences could be attributed to differences in the functional behavior of the different mutations. Epigenetic factors may also have a role for the observed phenotypic variability in different families.

The p.V42M substitution observed in the present cataract family represents a conservative amino acid change as both valine and methionine are uncharged non-polar amino acids. The other two missense mutations, p.G18V and p.S39C, also represent conservative amino acid changes. They are all located in exon 2, which constitutes domain 1 of CRYGS, and are obviously required for the maintenance of the tertiary structure of the protein and the morphology of the cortical fiber cells [14,28]. Thus, replacement of the structurally conserved valine-42 by methionine may disrupt the proper folding of the CRYGS polypeptide, leading to lens opacity. In vitro, γS-crystallin has an essential role in suppressing the aggregation of other γ-crystallin [29] and in interacting with α-crystallin [30]. We are unable to establish a clear genotype-phenotype correlation for the different mutations in γ-S crystallin linked with different cataract types. Functional studies of these mutations may explain the observed phenotypic differences.

In summary, we describe a novel missense mutation, p.V42M, in CRYGS that causes congenital cataract in seven members in a three-generation north Indian family. The phenotype differs from that of two other mutations in the same domain. Therefore, these findings further substantiate the clinical and genetic heterogeneity of dominant congenital cataract.
